# Differences between cancer patients and others who use medicinal Cannabis

**DOI:** 10.1371/journal.pone.0248227

**Published:** 2021-03-16

**Authors:** Matthew M. Cousins, Mary Jannausch, Reshma Jagsi, Mark Ilgen

**Affiliations:** 1 Department of Radiation Oncology, University of Michigan, Ann Arbor, Michigan, United States of America; 2 Department of Psychiatry, University of Michigan, Ann Arbor, Michigan, United States of America; 3 VA Center for Clinical Management Research (CCMR), Ann Arbor, Michigan, United States of America; University of Colorado Boulder, UNITED STATES

## Abstract

**Background:**

Cancer patients have been at the forefront of policy discussions leading to legalization of medical Cannabis (marijuana). Unfortunately, Cannabis use among those with cancer is poorly understood.

**Methods:**

A diverse group of patients seeking certification for medical Cannabis in the state of Michigan were surveyed at the time of their presentation to medical dispensaries. The survey assessed demographics, employment/disability, pain, physical functioning, mental health, mode of Cannabis use, and frequency/amount of Cannabis use. Chi-square and t-tests were performed to compare those who did and did not endorse cancer diagnosis.

**Results:**

Analysis of data from 1485 adults pursuing medical Cannabis certification, including 72 (4.8%) reporting a cancer diagnosis, indicated that those with cancer were older [mean age 53.4 years (SD = 10.5) vs. 44.7 years (SD = 13.0); p<0.001] than those without cancer. They also differed regarding employment status (p<0.001; working: 20.8% vs. 46.2%; disabled: 44.4% vs. 26.5% for those with vs. those without cancer, respectively). Those with cancer used less Cannabis (p = 0.033 for quantity used) and used Cannabis less often (p = 0.032 for frequency of use); they less frequently endorsed smoking Cannabis (80% vs 91%; p = 0.015). There was a non-significant trend to increased edible use in those with cancer (57% vs. 44%; p = 0.052).

**Conclusions:**

Patients with cancer who are seeking medical Cannabis are different from those seeking medical Cannabis without cancer, and they report using Cannabis differently. Further research to characterize the patterns and consequences of Cannabis use in cancer patients is needed.

## Introduction

Cancer is a universal qualifying condition for medical Cannabis across states providing access to medical Cannabis, and many patients with cancer use Cannabis to manage symptoms caused by either malignancy itself or the treatment of malignancy [1]. Patients with cancer report that they use Cannabis to help with pain, nausea, poor appetite, depression, anxiety, insomnia, and cancer itself, and some reports of observational studies have demonstrated perceived benefit of Cannabis for many symptoms [1–3]. Despite the broad range of patient goals that drive Cannabis use, there is a dearth of high quality evidence demonstrating that members of this population benefit from Cannabis use for many of these symptoms [4], and misinformation is a concern with regard to claims that Cannabis can cure cancer [5]. Additionally, some studies of Cannabis have specifically excluded patients with cancer [6]. As a result of these shortcomings in scientific understanding, there have been many calls for more research on Cannabis use in those with cancer, and recent funding announcements from federal agencies suggest that a greater emphasis is now being placed on this area by policy makers [4].

There are well understood differences between those with cancer or history of cancer and the overall population. For example, patients with a history of cancer suffer from greater disability and frequently report deficits in ability to carry out activities of daily living [7]. Individuals with cancer often must undergo complicated treatments to address their disease which may necessitate additional support [7]. The long term impact of these treatments can be severe and last the entire life of the patient. There may be significant worry associated with both initial treatment and over a potential long course of follow up after treatment is completed [7]. Despite these clear differences between cancer patients and others, it is not known how these differences may or may not translate into differences in Cannabis use between those with cancer and those using Cannabis for other medical reasons.

Given ongoing efforts to study many aspects of Cannabis use in those with cancer and continuously developing Cannabis policy, there is a need for basic information about Cannabis use in this population. Clinical trial designers need information on product preference and use characteristics to shape interventions for planned studies. Regulators need demographic information and Cannabis use details to aid in targeting of educational tools and labeling efforts to populations of patients with cancer. Clinicians need information about patients with cancer who use Cannabis and use patterns in this population as they attempt to thoughtfully consider benefits and risks in the context of frequently emerging new data. The present study is designed to provide insight of interest to these groups. We hypothesized that patients using Cannabis for cancer-associated symptoms are different from patients using Cannabis for non-cancer medical purposes in terms of their demographics, functional status, symptom severity, and Cannabis use patterns. This question was studied with a survey-based approach at multiple sites in a state with then medical-only Cannabis use where patients had access to a wide range of Cannabis and Cannabis-derived products.

## Materials and methods

### Subject recruitment

The process for recruitment and study design has been described previously [8]. Adults seen in four medical Cannabis clinics across Michigan for clinician certification for receipt or renewal of a medical Cannabis card between 01/2014 and 06/2015 were surveyed on a University of Michigan Institutional Review Board-approved protocol (HUM000661240 [8]) after providing written informed consent. The study was conducted in accordance with the Declaration of Helsinki. At the time, medical Cannabis use was legal in Michigan whereas recreational use was not. Medical Cannabis certification in Michigan is acquired through a process that includes (1) completing the patient portion of a form from the Michigan Marijuana Regulatory Agency (MRA, www.michigan.gov/MRA), (2) having a physician complete the provider component of the same form, and (3) submitting this form to MRA for review and approval. The law required individuals to have at least one qualifying condition for the use of medical Cannabis. Cancer was listed as a qualifying condition and use could be for the management of the symptoms or side-effects of cancer treatments. Products available for purchase were labeled with the weight of the purchased material or preparation as well as the concentration or amount of cannabidiol (CBD) and tetrahydrocannabinol (THC).

### Survey instrument

A paper survey instrument was administered as part of a previously described study as noted above. This survey assessed age, gender, race, educational attainment, relationship status, employment/disability, pain score via numeric rating scale (NRS) [9], physical functioning (PCS; Physical Component Summary) and mental health (MCS; Mental Component Summary via the Short Form-12 Health Survey; SF-12 [10, 11]), frequency of Cannabis use in the past 6 months, mode of Cannabis use in the last month, average amount of Cannabis used per week in the last month, number of hours spent feeling high per day in the last month, route of administration in the last month, and whether or not Cannabis use had been discussed with the individual’s primary care provider. Cancer diagnosis history and all other information was collected by self-report as part of the survey.

### Data analysis

Chi-square and t-tests were performed to assess differences between those who did and did not endorse a cancer diagnosis in terms of demographic factors, SF-12 metrics, and Cannabis use characteristics (p-value < 0.05 considered significant). All analyses were performed with SAS 9.4 (SAS Institute, Cary NC, USA).

## Results

### Demographics

A total of 1485 adults seeking medical Cannabis certification were surveyed, identifying 72 (4.8%) with a cancer diagnosis and 1413 (95.2%) without (Table 1). Those with cancer had a mean age of 53.4 years (standard deviation [SD] = 10.5) versus 44.7 years (SD = 13; p<0.001) among those without cancer. No differences were noted between those with cancer and those without cancer in terms of gender, race, relationship status, and education. Cancer patients were less than half as likely to be working full or part time (20.8% versus 46.2%), and they were more likely to be disabled (44.4% versus 26.5%; p<0.001) compared to medical Cannabis patients without cancer.

**Table 1:** Demographic and health characteristics of individuals seeking medical Cannabis certification for cancer versus not for cancer.

Characteristic	Seeking for Cancer		p-value ^a^
	Yes	No	
N (%)	72 (4.8%)	1413 (95.2%)	–
Age [years; Mean (standard deviation)]	53.4 (10.5)	44.7 (13.0)	<0.001
Male gender	39 (54.2%)	811 (57.4%)	0.59
White race	59 (81.9%)	1168 (82.7%)	0.88
Stable relationship	37 (51.4%)	782 (55.3%)	0.51
At least some college education	51 (71.8%)	908 (65.0%)	0.24
First time applicant for certification	34 (47.2%)	454 (33.2%)	0.02
Employment ^b^	–	–	<0.001
• Regular full/part time ^c^	15 (20.8%)	653 (46.2%)	–
• Disabled	32 (44.4%)	374 (26.5%)	–
• All others	25 (34.7%)	386 (27.3%)	–
Pain score per NRS [Mean (standard deviation)]	5.5 (2.7)	6.5 (2.0)	<0.001
SF-12 PCS [Mean (standard deviation)]	35.6 (7.8)	34.9 (8.2)	0.51
SF-12 MCS [Mean (standard deviation)]	40.5 (12.2)	45.8 (11.6)	<0.001
SF-12 MCS disability category ^b^	–	–	0.01
• Moderate to severe	29 (45.3%)	403 (30.2%)	–
• Mild to none	35 (54.7%)	931 (69.8%)	–

Abbreviations: NRS–numeric rating scale; SF-12 –Short Form-12 Health Survey; MCS–Mental Component Summary of SF-12; PCS–Physical Component Summary of SF-12.

^a^All tests of statistical significance were two-sided. Dashes in this column represent cells where comparison is inappropriate or relevant statistical test has been performed at the top of the relevant table section.

^b^Dashes represent cells in the title row.

^c^Includes those who reported disability and having regular employment of some kind.

### Symptoms and functioning

Relative to other medical Cannabis patients, cancer patients had less severe pain, with mean 5.5/10 (SD = 2.7) versus 6.5/10 (SD = 2.0; p<0.001) in non-cancer patients. Those with cancer were not different from those without cancer in terms of the physical component summary (PCS) from the SF-12. Mental component summary (MCS) assessment revealed that patients with cancer were more likely to be moderately to severely disabled (45.3%) based on this domain than those without cancer (30.2%).

### Cannabis use characteristics

**Fig 1:**
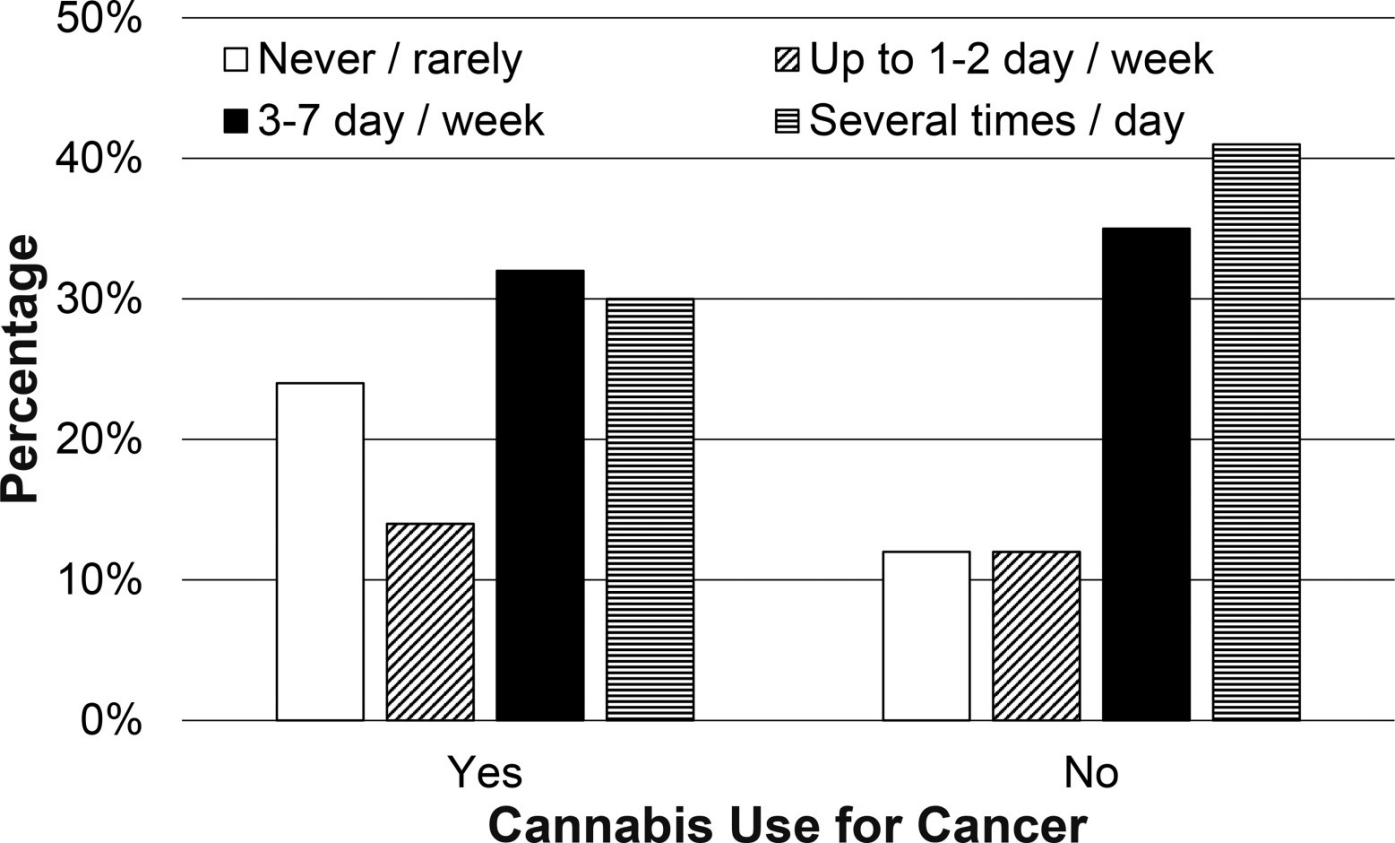
Frequency of medical Cannabis use. Reported frequency of Cannabis use for those who did and did not endorse cancer diagnosis.

**Fig 2:**
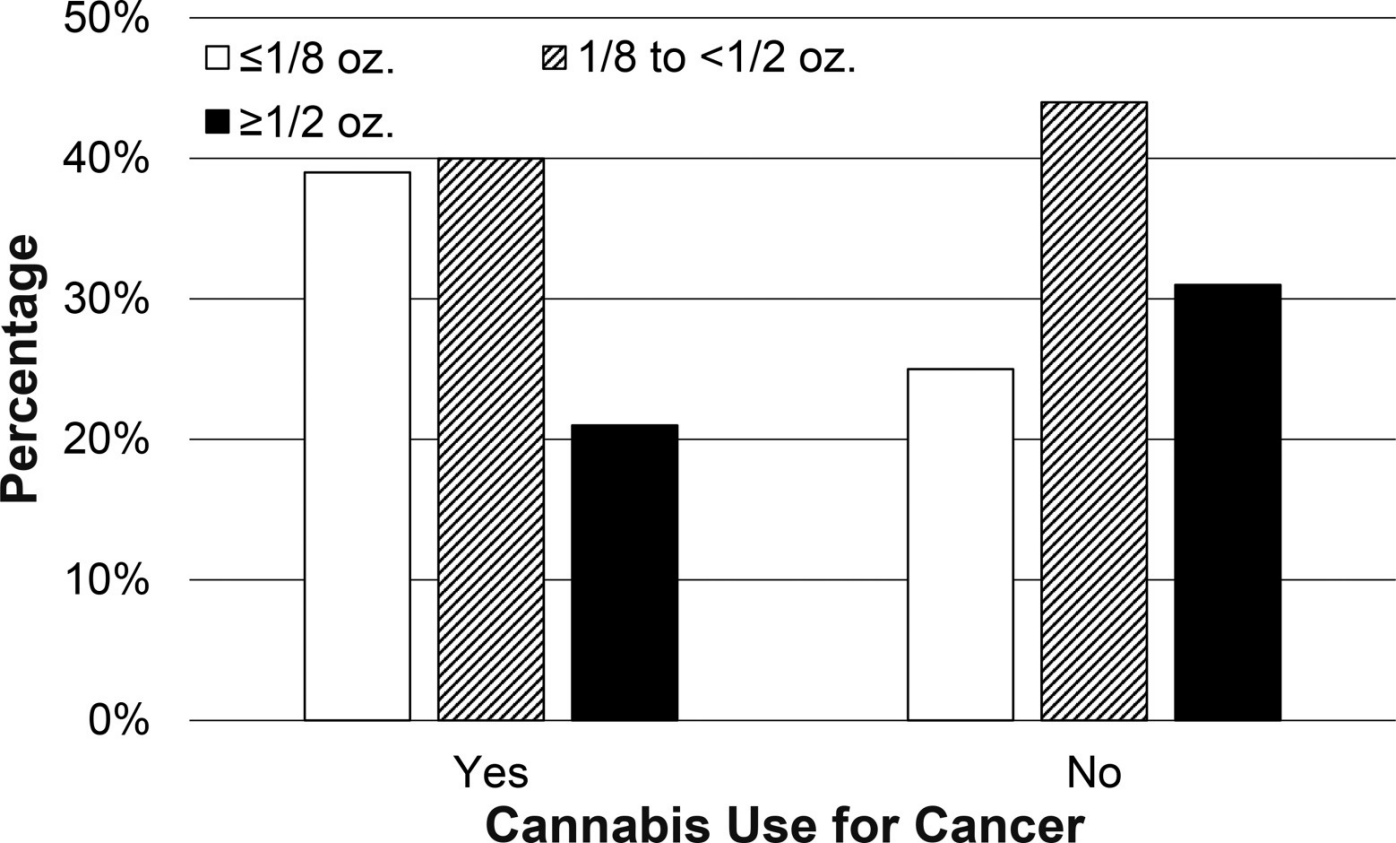
Quantities of Cannabis consumed. Amount of Cannabis consumed per week for those who did or did not endorse cancer diagnosis.

**Fig 3:**
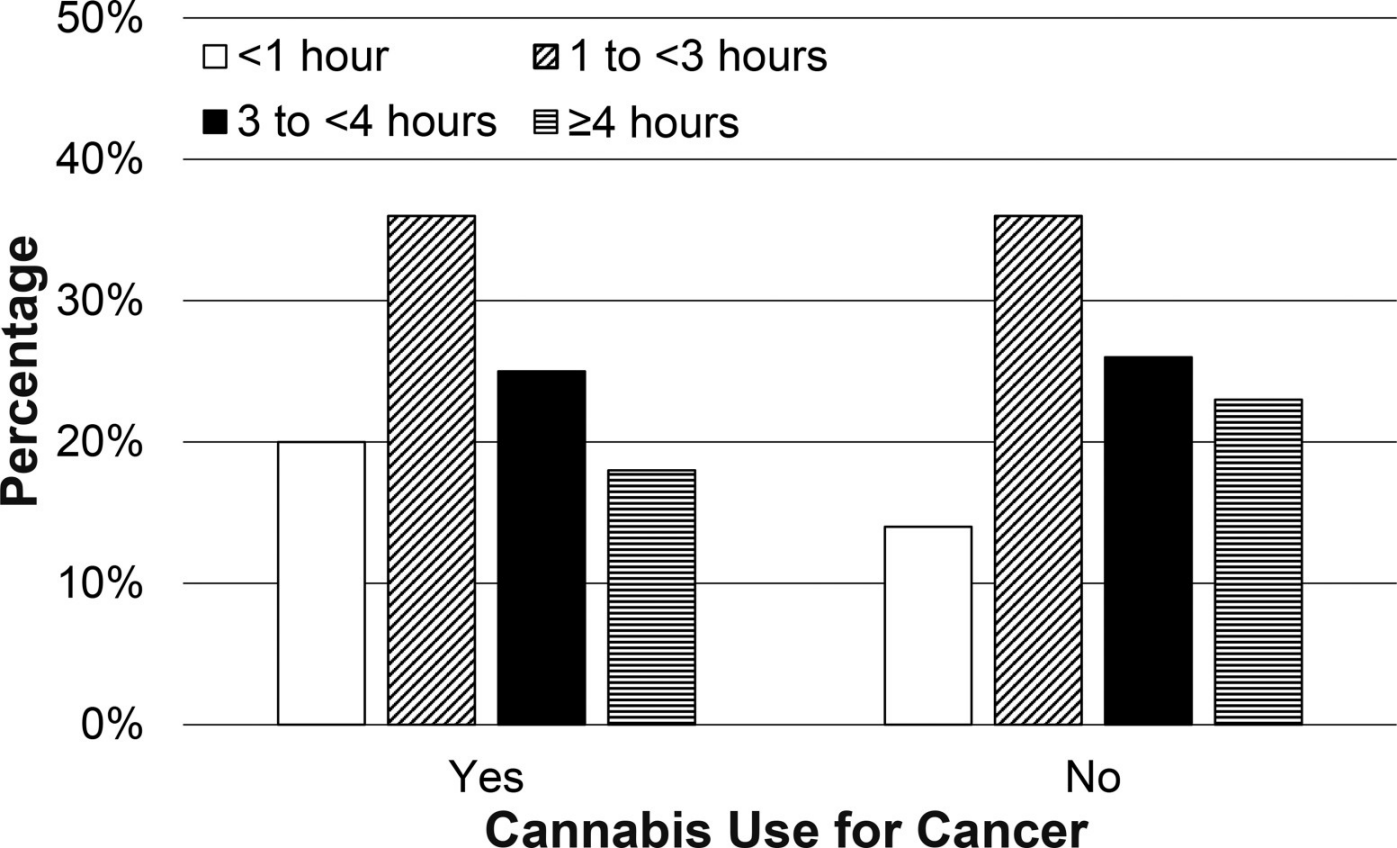
Time spent feeling high. Time per week spent feeling high for those who did or did not endorse cancer diagnosis.

In terms of medical Cannabis use frequency and amounts, cancer patients were twice as likely to endorse using Cannabis “none or rarely” in the last 6 months (23.6% versus 11.8%). Fewer cancer patients noted at least weekly Cannabis use (76.4% versus 87.6%) or use several times/day (30.5% versus 41.2%; p<0.05; Fig 1, S1 Table). Cancer patients used smaller quantities of Cannabis than those without cancer (p<0.05; Fig 2, S1 Table), with 38.6% of those with cancer reporting that they used <1/8 oz in the last week (compared to 24.9% of those without cancer). Nearly a third (31.3%) of those without cancer reported that they used >1/2 oz of Cannabis in the last week, while approximately a fifth (21.4%) of those with cancer reported using this amount of Cannabis in the same period. There were no statistically significant differences in amount of time spent feeling high or stoned in those with cancer versus those without cancer (Fig 3; p = 0.59).

**Fig 4:**
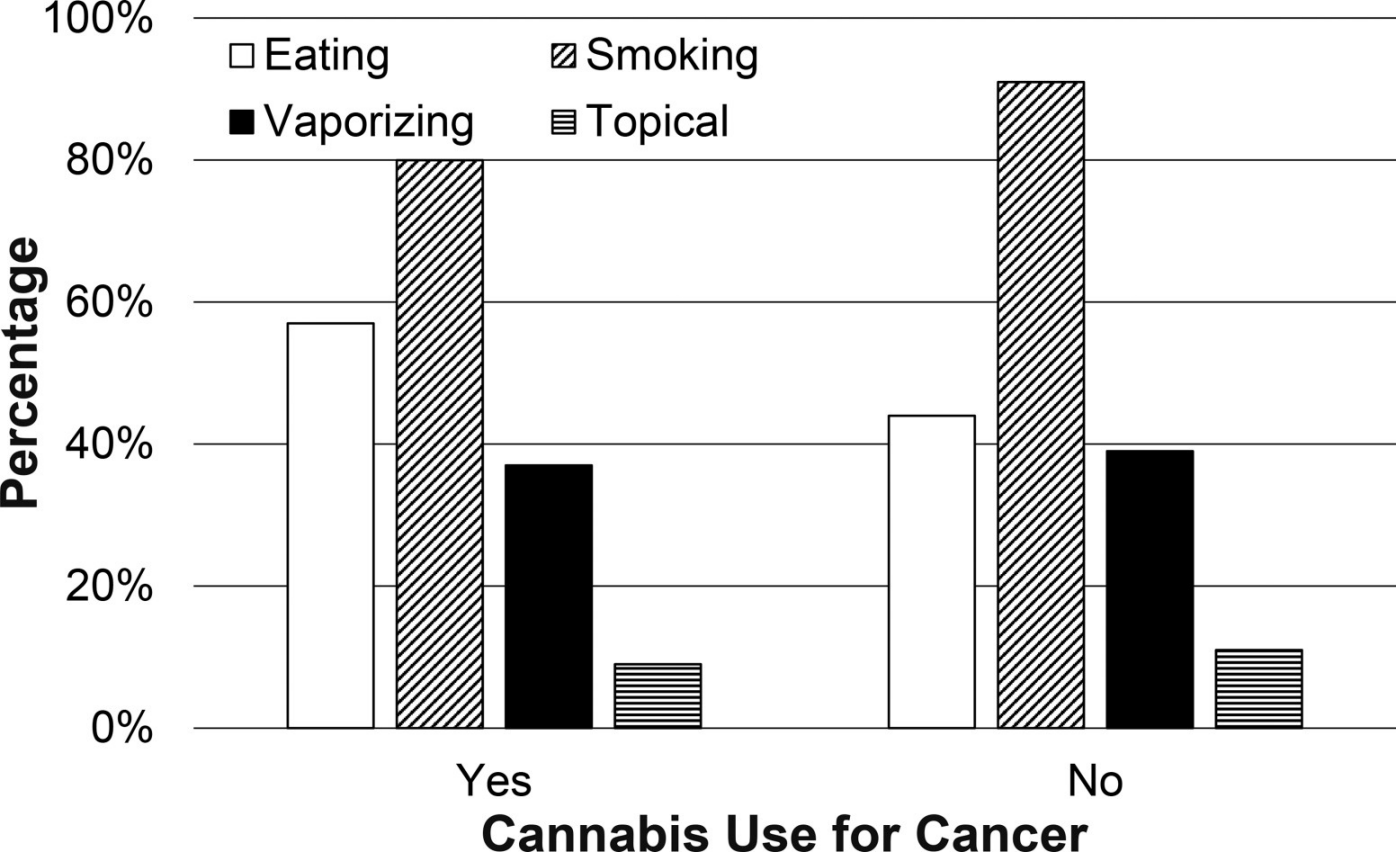
Cannabis mode of administration. Mode of Cannabis administration endorsed by individuals with and without cancer.

With regard to mode of administration, those with cancer were less likely to smoke Cannabis than other medical Cannabis patients (80.4% versus 91.2%; p = 0.015; Fig 4, S1 Table). There was also a non-significant trend towards increased oral administration of Cannabis, with 57.1% of those with cancer versus 43.9% of those without cancer endorsing use via this route (p = 0.05). There were no differences in numbers of individuals administering Cannabis through vaping (37% vs. 39%; p = 0.85) or topical administration (9% vs. 11%; p = 0.62).

### Certification and physician discussion

Individuals with cancer were more likely to lack an active medical Cannabis certification (47.2% versus 33.2%; p = 0.02), although we do not know what proportion of these individuals were applying for certification renewal vs. first time certification. It is important to note that individuals without an active medical Cannabis certification were less likely to endorse Cannabis use if they had cancer than if they did not have cancer (p<0.05). Among those with cancer, 53.8% had discussed Cannabis use with their primary care doctor in the last 6 months while 44.9% of those without cancer endorsed a similar discussion (p = 0.2).

## Discussion

Differences between patients with cancer and those who do not have cancer who are seeking Cannabis are not well understood, but more information on the differences between these two groups is needed to support ongoing research efforts and policy decisions. Using a survey-based approach, we characterized groups of adults seeking medical Cannabis certification who did and did not have cancer. These data revealed that adults with cancer have multiple features that distinguish them from other adults seeking medical Cannabis certification. These differences fall into four main areas: (1) demographics, (2) symptoms and functioning, (3) Cannabis use characteristics (frequency/amount and mode of administration), and (4) certification-related differences. We will review each of these in the following paragraphs.

Several demographic differences were noted in this study. These included the finding that those with cancer were older than those without cancer, in agreement with prior findings from a single dispensary in New York [12]. Given that cancer diagnoses increase with age, this finding is not surprising. Additionally, those with cancer were less likely to be working and more likely to be classified as disabled. Both of these findings would be expected to be associated with both age and potential symptoms from cancer diagnosis. We did not note differences in gender, race, or educational attainment between those with cancer and those without cancer, suggesting that the populations were relatively balanced in terms of these factors. Others have noted female predominance previously in those with cancer, though there is a general male predominance amongst those using Cannabis in the general population and in those with cancer [12–14]. Future studies should carefully consider age and employment status when seeking to understand Cannabis use in cancer patients given demographic differences observed in this study.

Symptom and functioning differences between those with cancer and those without cancer are considered together, as they are likely closely linked. In terms of symptoms, patients with cancer generally had similar physical function to those without cancer as assessed by PCS in setting of less severe pain. It is important to note that 92% of those without cancer were seeking Cannabis for pain (similar to previous reports), suggesting that the non-cancer population was enriched for those with severe pain [12]. This may explain why those with cancer had less pain than those without cancer. People with cancer also noted greater mental health-related disability as measured by MCS from the SF-12, a finding that is of particular concern given conflicting reports as to the impact of Cannabis on mood and mental health as well as a Canadian report that noted higher suicidal ideation among cancer patients using Cannabis [4, 14]. Others have suggested that Cannabis may benefit head and neck cancer patients through reduction in anxiety and pain and improved quality of life [15]. The mental health difficulties associated with cancer diagnosis and treatment are well known, and a better understanding of mental health implications of Cannabis use in this vulnerable population will be key as researchers seek to understand risk and benefits of Cannabis use in those with cancer [16, 17].

Individuals with cancer used Cannabis less frequently and in smaller amounts than those without cancer. Specifically, more than one fifth of those with cancer reported never/rarely using Cannabis. Given the prominent role that patients with cancer have played in policy discussions, it was expected that those with cancer would use Cannabis more frequently. A possible explanation for at least some of the lower observed usage rates in patients with cancer may be related to differences in certification rates. Those with cancer had lower certification rates, and cancer patients were less likely to endorse Cannabis use if they lacked certification. Taken together, lower prior certification rates and what appears to be better adherence to legal guidance suggests that policy and/or regulatory changes may differentially impact those with cancer relative to other medical Cannabis patients. Individuals with cancer were also older than others, and there are known to be age-related differences in Cannabis use that may relate to perceptions about Cannabis [18]. This could also potentially explain differences, though large scale data on Cannabis perception are limited in those with cancer.

Those with cancer had different route of administration preferences. Fewer cancer patients chose to smoke Cannabis. Additionally, there was a nearly significant increase in the use of edible preparations in those with cancer with a p-value of 0.052. Further characterization of these differences and what drives them will be important to better understand this population of patients using Cannabis and guide educational and supportive policies and practices. Given that there were no differences with regard to the proportion of individuals who discussed Cannabis use with their primary care provider, it seems less likely that primary care provider recommendations against smoking might have played a role, but it is important to note that some groups of providers have recommended against smoking Cannabis [19].

As noted above, there were no differences in the proportion of patients who told their doctor about their Cannabis use between those with cancer and those without cancer. Approximately half of individuals with cancer noted that they had spoken with their doctor. We have no evidence as to the amount of education that individuals might have received in setting of these conversations, but others have noted that little advice is given by providers when Cannabis use is revealed [20]. Available data suggest that much patient education on Cannabis comes from outside of the medical community [20].

There are a number of key weaknesses of this study that should be recognized. The small size of this study makes generalizations to larger populations of medical Cannabis users less reliable and likely obscures some additional potential differences. The finding of less pain in cancer patients and equivalent physical functioning could be the result of bias due to the location of recruitment at ambulatory dispensaries and the requirement that a participant have the ability to fill out a survey. Those with advanced, painful bony metastatic disease might be under-represented in this setting. With regard to use frequency data, there is confounding between medical Cannabis card possession (lower in those with cancer) and cancer diagnosis. This likely did not impact other analyses beyond the use frequency and amount analyses. We would note that the concept of a “high” related to Cannabis may mean different things to different groups of individuals that could impact interpretation given that these data were collected by self-report. We also did not capture Cannabis product potency, so it is not possible to draw conclusions with regard to concentration of particular active compounds in products utilized by different groups. Despite these limitations, these data provide valuable insight into a very poorly studied area.

## Conclusions

Overall, these analyses demonstrate that individuals with cancer are somewhat different from the general population of individuals using medical Cannabis in terms of demographics, symptoms and functioning, Cannabis use patterns, and certification status. This work highlights the need to better understand Cannabis use in cancer patients under real-world conditions, as suggested by others [4]. A better grasp of unique features of individuals with cancer who use Cannabis will be very important as researchers, clinicians, and policy-makers seek to understand the ways in which Cannabis use relates to symptoms and functioning over time. Therefore, the findings detailed in this report support the study of Cannabis use specifically within cancer patients, a group that is understudied despite their prominent role in policy discussions on the topic of medical Cannabis.

## Supporting information

S1 Table Characteristics of Cannabis use among those seeking medical Cannabis for cancer and those seeking Cannabis for other reasons. (PDF)
