# Supplementary material for: Differences between cancer patients and others who use medicinal Cannabis
Source: PLoS One. 2021 Mar 16;16(3):e0248227. doi: 10.1371/journal.pone.0248227 (PMC7963092; doi:10.1371/journal.pone.0248227)
Supplement: S1 Table — (PDF) [file pone.0248227.s001.pdf]

Differences between cancer patients and others who use medicinal Cannabis

Matthew M. Cousins[1], Mary Jannausch[2], Reshma Jagsi[1], and Mark Ilgen[2,3]

[1]Department of Radiation Oncology, University of Michigan, Ann Arbor, Michigan, United States of America
[2]Department of Psychiatry, University of Michigan, Ann Arbor, Michigan, United States of America
[3]VA Center for Clinical Management Research (CCMR), Ann Arbor, Michigan, United States of America

S1 Table. Characteristics of Cannabis use among those seeking medical Cannabis for cancer and those seeking Cannabis for other reasons.

**Cannabis use characteristic**	**Seeking for Cancer**		**p-value** [a]
	**Yes**	**No**	
Medical Cannabis use [b,c]	–	–	0.032
-None to rarely	17 (24%)	165 (12%)	–
-Up to 1-2 days / week	10 (14%)	164 (12%)	–
-3-7 days / week	23 (32%)	496 (35%)	–
-Several times / day	22 (30%)	578 (41%)	–
Amount Cannabis used / week [b,d]	–	–	0.033
-None / < 1/8 oz.	27 (39%)	341 (25%)	–
-1/8 to < ½ oz.	28 (40%)	600 (44%)	–
-½ oz. or more	15 (21%)	429 (31%)	–
Hours / day feeling high [b,d]	–	–	0.59
-Less than 1 hour	11 (20%)	174 (14%)	–
-1-2.99 hours	20 (36%)	454 (36%)	–
-3 to 3.99 hours	14 (25%)	333 (26%)	–
-4+ hours	10 (18%)	293 (23%)	–
Modes of Use [b,e]	–	–	–
-Eating / ingesting	32 (57%)	555 (44%)	0.052
-Smoking	45 (80%)	1153 (91%)	0.015
-Vaping	21 (37%)	490 (39%)	0.85
-Skin/topical	5 (9%)	139 (11%)	0.62

[a] All tests of statistical significance were two-sided. Dashes in this column represent cells where comparison is inappropriate or relevant statistical test has been performed at the top of the relevant table section.
[b] Dashes represent cells in the title row.
[c] Past 6 months.
[d] Past month.
[e] Total number of responses for mode of administration questions was 1320 of 1485. No response was obtained for these items for 165 individuals (140 due to no Cannabis use in the last month; 25 refused to answer). Of the 1320 who responded, 56 endorsed cancer diagnosis and 1264 did not.
